# Effects of Bicarbonate Stress on Serum Ions and Gill Transporters in Alkali and Freshwater Forms of Amur Ide (*Leuciscus waleckii*)

**DOI:** 10.3389/fphys.2021.676096

**Published:** 2021-09-14

**Authors:** Yu Mei Chang, Xue Fei Zhao, Hon Jung Liew, Bo Sun, Shuang Yi Wang, Liang Luo, Li Min Zhang, Li Qun Liang

**Affiliations:** ^1^National and Local Joint Engineering Laboratory for Freshwater Fish Breeding, Heilongjiang River Fisheries Research Institute, Chinese Academy of Fishery Sciences, Harbin, China; ^2^College of Wildlife and Protected Area, Northeast Forestry University, Harbin, China; ^3^Higher Institution of Center Excellence, Institute of Tropical Aquaculture and Fisheries, Faculty of Fisheries and Food Science, Universiti Malaysia Terengganu, Kuala Nerus, Malaysia; ^4^College of Fisheries and Life Science, Shanghai Ocean University, Shanghai, China

**Keywords:** amur ide (*Leuciscus waleckii*), alkaline water, bicarbonate alkalinity, environment stress, ionoregulation, gill transporters, bicarbonate extrusion

## Abstract

The Amur ide (*Leuciscus waleckii*) is a fish in the Cyprinidae family. Compared with other Amur ide living in freshwater ecosystems, the Amur ide population in Lake Dali Nor of China is famous for its high tolerance to the alkaline conditions of 54 mM (pH 9.6). Yet, surprisingly, the ionoregulatory mechanism responsible for this remarkable alkaline adaptation remains unclear. Therefore, this study sought to investigate how bicarbonate affects the acid-base balancing and ionoregulatory responses of this animal. Here, using a comparative approach, the alkali form of Amur ide and its ancestral freshwater form living in other freshwater basins were each exposed to 50 mM (pH 9.59 ± 0.09), a level close to the alkalinity of Lake Dali Nor, and their physiological (AE1) adjustment of ions and acid-base regulation were investigated. This study highlighted differences in blood pH and serum ions (e.g., Na^+^, K^+^, Cl^−^, and Ca^2+^), Na^+^/K^+^ ATPase (NKA) activity and its mRNA level, and mRNA expression of gill transporters (Na^+^/H^+^ exchanger member 2 and/or 3, Na^+^/${\text{HCO}}_{3}^{-}$ cotransporter (NBC1), Cl^−^/${\text{HCO}}_{3}^{-}$ exchanger, Na^+^/Cl^−^ cotransporter (NCC), Na^+^/K^+^/2Cl^−^ (NKCC1), SLC26A5, and SLC26A6) for alkalinity adaptation between the two forms of Amur ide differing in alkalinity tolerance. Specifically, close relationships among the serum Na^+^ and mRNA levels of NCC, NKCC1, and NHE, and also NKA and NBC1, in addition to serum Cl^−^ and bicarbonate transporters (e.g., SLC26A5 and SLC26A6), characterized the alkali form of Amur ide. We propose that this ecotype can ensure its transepithelial Cl^−^ and Na^+^ uptake/base secretions are highly functional, by its basolateral NKA with NBC1 and apical ionic transporters, and especially NCC incorporated with other transporters (e.g., SLC26). This suggests an evolved strong ability to maintain an ion osmotic and acid-base balance for more effectively facilitating its adaptability to the high alkaline environment. This study provides new insights into the physiological responses of the alkaline form of the Amur ide fish for adapting to extreme alkaline conditions. This information could be used as a reference to cultivating alkaline-tolerant fish species in abandoned alkaline waters.

## Introduction

Amur ide (*Leuciscus waleckii*), which belongs to the Cyprinidae, is an economically important native species mainly distributed in the Amur River, Liao River, Yellow River, and inland lakes in northern parts of China. This species serves as an economical protein source for people living in the northern region of China who are far away from marine resources, and eventually become an economically important species for local fishery (Chi, 2010). Ecologically, Amur ide contributes to providing nutrients to balance the ecosystem, especially for birds migrating from Siberia to the South as a food supply (Xu et al., 2017). Due to its importance in human life and ecology, attention has been focused on Amur ide and discovered that one of the Amur ide population that live in Lake Dali Nor (43°22′43′′ N, 116°39′24′′ E) is known for its high tolerance to alkaline water (AW) (Xu et al., 2017; Chen et al., 2019; Wang et al., 2021; Zhao et al., 2021). The Lake Dali Nor is a carbonate alkali-saline lake with a water pH of about 9.6 or 54 mM alkalinity (${\text{HCO}}_{3}^{-}$/${\text{CO}}_{3}^{2-}$ concentrations) and salinity of about 6‰ (Chi, 2010; Chang et al., 2013b).

To live in alkali-saline Lake Dali Nor, the Amur ide must equip special ionoosmoregulation and acid-base regulation effectively to adjust and maintain internal homeostasis (Fielder et al., 2007; Evans, 2010; Kidder et al., 2010; Al-Jandal and Wilson, 2011) by modulating a net flux Na^+^ and Cl^−^ movement across the gill epithelium (Wilkie and Wood, 1996). However, the physiological adaptation mechanisms of this alkaline form of Amur ide, which live in the alkaline-saline Lake Dali Nor, remains unclear. We recently discovered 21 potential genes that are involved in osmoregulation such as DLG1, VIPR1, AKT1, and GNAI1 (Wang et al., 2021). Long non-coding RNAs (lncRNAs) are expressed in the gills and kidneys, which are associated with osmoregulation and metabolism-related pathways that maintain the homeostasis of alkali-saline form Amur ide (Zhao et al., 2021). Of all information discovered above, functional ionotransporters activities in alkali-saline form Amur ide remain unknowns. To our knowledge, literature regarding fundamental physiology in the osmoregulation aspect has yet to be revealed. Therefore, it is important to understand how this alkali-saline form Amur ide maintains balance homeostasis under high alkaline conditions.

There are a few species investigated for how freshwater fishes adapt and survive in alkali-saline water. For example, Lahontan cutthroat trout modified freshwater-type chloride cell apical fractional surface area to adjust net fluxes OH^−^, ${\text{HCO}}_{3}^{-}$, and ${\text{CO}}_{3}^{2-}$ electrochemical potential gradients facilitated by Cl^−^/${\text{HCO}}_{3}^{-}$ transporter to mediate base excretion in Pyramid Lake (Wilkie et al., 1994). Rainbow trout cultured under high pH water regulates Na^+^ and Cl^−^ movements in gill epithelium to regulate acid-base balance accompanied by net fluxes of H^+^ and base ${\text{HCO}}_{3}^{-}$ or OH^−^. This achievement was reported in facilitating Cl^−^/${\text{HCO}}_{3}^{-}$ and Na^+^/H^+^ exchange mechanisms (Mcdonald and Prior, 1988; Goss et al., 1992). Another unique species Magadi tilapia, lives in Lake Magadi, with pH 10 was discovered to uses modified seawater-type chloride cells (Pierre et al., 2000). It is believed that these modified seawater-type chloride cells facilitate Na^+^ and Cl^−^ excretion *via* basolateral Na^+^/2Cl^−^/K^+^ cotransporter for Cl^−^ and Na^+^/K^+^ ATPase (NKA) for Na^+^ excretion. While, base excretion is facilitated by the combination of Cl^−^/${\text{HCO}}_{3}^{-}$ antiporter and ${\text{HCO}}_{3}^{-}$/${\text{CO}}_{3}^{2-}$ transport system to maintain internal Cl^−^ balance (Wood and Bergman, 1994). ${\text{HCO}}_{3}^{-}$ excretion is linked to Cl^−^ uptake and/or Na^+^ excretion and correspondingly, the Cl^−^/${\text{HCO}}_{3}^{-}$ exchanger (AE1, SLC26 family) and/or Na^+^/${\text{HCO}}_{3}^{-}$ cotransporter (NBC1), which suggests their involvement in branchial bicarbonate transport in other fish species (Lee et al., 2011a; Boyle et al., 2015; Michael et al., 2016; Ruiz-Jarabo et al., 2017).

From the physiological perspective to living in an extreme environment, effective gills phenotypic plasticity is necessary to cope with the unfavorable conditions especially on the ionoosmoregulation (Mohamad et al., 2021). For example, in freshwater fishes, NKA plays a key role in maintaining high K^+^ and low Na^+^ gradients in ionocytes as a driving force to facilitate other transmembrane transporter activities (Hirose et al., 2003; Guo and Sun, 2005; Liew et al., 2013). Therefore, the NKA activity, NKA enzyme availability, and NKA gene expression are often suggested as reliable osmoregulatory indicators in freshwater fish (Deane and Woo, 2005; Evans, 2005; Tsai et al., 2018). Ionoregulatory metabolon revealed the NKA functioning in cooperation with apical transporters, such as Na^+^/H^+^ exchanger member 2 and/or 3 (NHE2/3) (Inokuchi et al., 2008; Liu et al., 2013), the V-type proton ATPase (Horng et al., 2007; Liew et al., 2015), the Na^+^/Cl^−^ cotransporter (NCC) (Hiroi et al., 2008; Wang et al., 2009; Chang et al., 2013a; Kwong and Perry, 2016), Na^+^/K^+^/2Cl^−^ (NKCC1) (Hiroi and Mccormick, 2012), and the cystic fibrosis transmembrane conductance regulator to regulate ion transportation through transepithelial (Hwang and Lee, 2007; Marshall, 2011).

Together with all the ionoregulation information as background, it is important to reveal ionoregulaory mechanisms of Amur ide to adapt to the extreme alkali-saline environment, specifically to understand how Amur ide regulate acid-base balancing and transporter involves. Therefore, this study was designed with an aim to investigate the effect of bicarbonate on acid-base and ionoregulatory responses of Amur ide exposed to bicarbonate AW. To address this objective, the freshwater-form Amur ide was selected as a control and compares with the alkaline-form Amur ide exposed to 50 mM bicarbonate AW condition. Through alkaline adaptability, this information can be used as basic knowledge for selective breeding to develop alkaline-tolerant strain Amur ide and promote utilization of the abandoned AW effectively for aquaculture purposes.

## Materials and Methods

### Ethics Statements

In this study, all the animal procedures were performed according to the *Guidelines for Care and Use of Laboratory Animals* of the Heilongjiang River Fisheries Research Institute of the Chinese Academy of Fishery Sciences.

### Source of Fish Individuals and Their Management

A total of 120 F1 of the ancestral freshwater-form Amur ide juveniles from the Songhua River (SH) and 120 F3 of the alkaline-form Amur ide juveniles from Lake Dali Nor (DL) were collected from the Hulu Experimental Station of the Heilongjiang River Fisheries Research Institute (126.63°E, 45.97°N). All the fishes in this station were maintained in outdoor high-density polyethylene ponds. All juveniles collected for this study were at the age of 3-month old with average body weight (BW) at 48.72 ± 6.89 g and body length at 14.61 ± 1.33 cm, respectively. Selected specimens were transferred to an indoor rearing facility at Heilongjiang River Fisheries Research Institute, SH and DL specimens were distributed equally into two 650-L rearing aquariums with 120 fishes and allowed to acclimate to the aquarium conditions for a week before experimentation. Each aquarium was equipped with an individual external recirculating water system following water-quality specifications. Water qualities were measured by using YSI multiple water quality meter (YSI, Ohio, USA) to monitor the temperature (23.26 ± 0.52°C), dissolved oxygen level (8.24 ± 0.51 mg/l), pH (7.29 ± 0.05), salinity (0.14 ± 0.03 mg/l), and alkalinity (0.46 ± 0.02 mM). A total of 50% water volume was refreshed two times a day. During acclimation, all fishes were fed two times a day at a 1% BW with commercial pellets (Shandong Shengsuo Feed Technology Co, Ltd, China), and uneaten food, within 15 min, was removed to avoid water deterioration. Fishes fasted for 48 h before experimentation.

### Experimental Design and Bicarbonate Exposure

This was experiment planned with a single factorial design consisted of two forms of Amur ide (DL and SH) exposed to 50 mM bicarbonate (NaHCO_3_) AW at different time-course intervals (day 1, 3, 5, and 7) in a 200-L aquarium. All treatments were set in triplication with a control group of each DL and SH form with 10 fishes in each replicate.

To impose the 50 mM bicarbonate alkalinity stress exposure, a total of 839 g of bicarbonate AW (Tianjin Kemiou Chemical Reagent Co Ltd, China) was dissolved in tap water after 24 h of aeration and added into each exposure aquarium. The bicarbonate concentrations in each replication were monitored daily with 0.02 mM HCl titration. Freshly prepared 50 mM bicarbonate AW was used for the daily water replacement at 50% after sampling. YSI water analyzer was used to monitor the water quality of each replicate and maintained as follows: temperature at 24.02 ± 1.28°C and 24.7 ± 0.95°C, salinity at 2.18 ± 0.09 mg/l and 2.15 ± 0.38 mg/l, dissolved oxygen at 11.74 ± 0.31 mg/l and 11.73 ± 0.05 mg/l, and pH at 9.63 ± 0.10 and 9.56 ± 0.12 with alkalinity at 52.84 ± 1.15 and 51.91 ± 3.81 mM, respectively.

### Fish Sampling

Sampling time-course intervals were set at 24 h (d1) followed by 48 h intervals at d3, d5, and d7 after exposure to 50 mM bicarbonate AW. At each sampling time course, three fishes were sampled from each replicate (three replicates, hence *n* = 9). Whereas for the control group, sampling was performed at the start (*n* = 4) and the end of the experiment (*n* = 5) denoted as d0. This is to reduce the number of fish from becoming scarified. During their sampling, all the fishes were anesthetized with a neutralized MS222 at 100 mg/l for about 30 s (Pharmaq Ltd, UK). This solution was prepared by using the same experimental water from their respective aquarium. Blood sample of each individual was collected *via* caudal peduncle using a 1 ml non-heparinized syringe; an aliquot of the blood was taken out for its pH measurement immediately and the rest of the blood was centrifuged immediately at 3,500 rpm for 10 min at 4°C (Deng et al., 2018). Serum extracted from each sample was immediately frozen in liquid nitrogen and stored at −80°C for later analysis. The left-side branchial lamellae samples per individual were excised, frozen in liquid nitrogen, and immediately stored at −80°C for NKA activity, and the right-side branchial lamellae samples per individual were collected for quantitative real-time PCR (RT-qPCR) analysis.

### Analytical Techniques

#### Blood pH and Serum Ions

Blood pH was measured immediately after the blood was sampled by using a pH meter (SevenCompact S210, Mettler Toledo, Switzerland). Before these measurements, pH and conductivity electrodes were calibrated with three pH standard buffer solutions at pH 4, 7, and 10. Each serum sample was diluted five times with ultrapure water (Millipore, MA, USA), and their ion concentrations were measured by atomic absorption spectrophotometry with a 4100 atomic absorption analyzer (PE Company, USA). Meanwhile, lanthanum was added to each serum sample for its Ca^2+^ measurement to avoid ion interference (Trudeau and Freier, 1967).

#### Gill Na^+^/K^+^ ATPase Activity

Gill samples in triplication were rapidly homogenized in liquid nitrogen, then suspended in 0.9% physiological saline water (w/v: 1/9) and inverted three to four times. Supernatants were obtained by centrifugation at 2,500 rpm, for 10 min at 4°C, and supernatants were used for the NKA enzymatic assay. Gill NKA activity was determined by following the instructions of the NKA enzyme kit (Nanjing-built Technology Co. Ltd., China) and measured by using a Bio-Teck Microplate Reader (Bioteck Instruments Inc, Vermont, USA), while the protein concentrations were measured with the Bradford reagent (Nanjing-built Technology Co. Ltd., China).

#### mRNA Expression of Ion Transporters

Total RNA from the gill samples was extracted by using the Trizol^®^ Reagent (Invitrogen, NY, USA), according to the instruction of the manufacturer. All the isolated total RNA was quantified spectrophotometrically, using a NanoDrop^TM^ 8,000 Spectrophotometer (Thermo Fisher Scientific, MA, USA). Followed by performing gel electrophoresis at a volume of ~200 ng into 1.5% agarose gel to assess the RNA integrity. First-strand cDNA was synthesized from each sample by using the PrimeScript^®^ RT reagent kit with the gDNA Eraser (TaKaRa, Dalian, China) according to the instructions of the manufacturer. The RT-qPCR was carried out using ABI 7,500 sequence analysis system (Applied Biosystems, CA, USA).

The respective sequences of candidate genes were extracted from the transcriptome data of Amur ide (Chang et al., 2014), for which the primer pairs were designed in Prime 5.0 (Table 1), the 18S ribosome RNA served as an internal control. A two-step RT-qPCR program was conducted as follows: enzyme activation step at 95°C for 1 min, followed by 40 cycles of 95°C for 5 s and 60°C for 34 s. The presence of a single product was confirmed by a melting curve analysis. Data were collected and analyzed using the 2^(−ΔΔCt)^ method (Livak and Schmittgen, 2002). The CT values for 18S rRNA did not change, following transfer to high alkalinity.

**Table 1:** Information on the primer pairs used to quantify expression of genes *via* RT-qPCR. RT-qPCR: quantitative real-time PCR.

**Protein ID**	**Annotation**	**Primer sequences (5'−3')**	**Size of products (bp)**
NKA α1-like	Na^+^/K^+^ ATPase alpha 1	F:GCTCTGCTACTTGGTCTG R:TGGTATTCTTGTGGACTGAG	249
NKA α3	Na^+^/K^+^ ATPase alpha 3	F:TCGGACAAGACTGGAACA R:CGGACTCAGAAGCATCAC	239
NHE2	Na^+^/H^+^ exchanger, SLC family 9, member 2	F:CCAGATCGAGGCTTTTGGTG R:ACAGCATCATTGAACAGGCA	171
NHE3b	Na^+^/H^+^ exchanger, SLC family 9, member 3	F:TCAATCCCATTTGTCCCGTT R:GTTTGACACCCATCTTAGCTGA	154
NCC	Na^+^/Cl^−^ cotransporter, SLC family 12, member3	F:TTCAATGCCGACAGTCTG R:CGTCCAATCCATCCATCAT	211
AE1	Anion exchanger, SLC family 4, member 1	F:GAAGGAGCGCATGAAGAAGG R:AGCTTCTTTCTCTCCACCCC	225
NKCC1	Na^+^/K^+^/2Cl^−^ cotransporter, SLC family 12, member 2	F:GATCAAGTCAGGTCAGTGT R:CCAGTCGGAATCTCTTCAA	220
SLC26A6	Cl^−^/${\text{HCO}}_{3}^{-}$ exchanger, SLC family 26, member 6	F:CCCCAAACCAACACAGACTG R:GCTGCGTTGACTCTGGATTT	181
SLC26A5	Cl^−^/${\text{HCO}}_{3}^{-}$ exchanger, SLC family 26, member 5	F:CGTCCTATCGTGCTACTGGT R:TTGCCAGTTCGATCTTGCTG	160
NBC1	Na^+^/${\text{HCO}}_{3}^{-}$ cotransporter, SLC family 4, member 4	F: AGCGGTCACTGGAGCCATCTT R:AGTTGCCACCAGGACCAGACAG	189
18S	18S rRNA	F:GGAGGTTCGAAGACGATCAG R:GTGAGGTTTCCCGTGTTGAG	183

### Statistical Analysis

The data were analyzed statistically using one-way ANOVA in SPSS 19.0 software (SPSS, Chicago, IL, USA). The least significant difference (LSD) test was used to assess the differences among sampling time points within a group. Student's *t*-test was used to assess the differences between DL and SH groups within the same sampling time point. Significance was set at *P* < 0.05. Data are presented here as the means ± SE.

## Results

### Blood pH and Serum Key Ions

Throughout the experimentation period, all fishes were survived till the end of the experiment. The blood pH of the two forms of Amur ide exposed to bicarbonate AW is shown in Table 2. Blood pH for SH fluctuated significantly when exposed to bicarbonate AW, but blood pH for DL was maintained relatively stable. This showed that DL performed better than SH in terms of alkaline adaptability. Throughout 7 days of bicarbonate AW exposure, we found that lower blood pH was noticed at d3 and d5 in SH at pH 7.92 ± 0.11 and 7.99 ± 0.07, respectively. At d7, the blood pH was recorded at 8.49 ± 0.70, which was the higher pH level indicated freshwater-form (SH) Amur ide experiencing bicarbonate disturbance exposed to AW.

**Table 2:** Effects of bicarbonate AW (NaHCO_3_) stress on the blood pH (*n* = 9) in DL (the alkali form introduced from Lake Dali Nor) and SH (the freshwater form introduced from Songhua River) fish forms.

**Sampling time**	**DL**	**SH**	**DL vs. SH *P* value**
Day0	8.00 ± 0.12^a^	8.07 ± 0.13^ab^	0.41
Day1	8.00 ± 0.16^a^	8.05 ± 0.27^ab^	0.55
Day3	7.85 ± 0.13^a^	7.92 ± 0.11^b^	0.37
Day5	8.03 ± 0.37^a^	7.99 ± 0.07^b^	0.81
Day7	8.10 ± 0.15^a^	8.49 ± 0.70^a^	0.31

*Values with the same letter within columns are not significantly different, whereas different letters indicate significant differences (P < 0.05)*.

**Figure 1:**
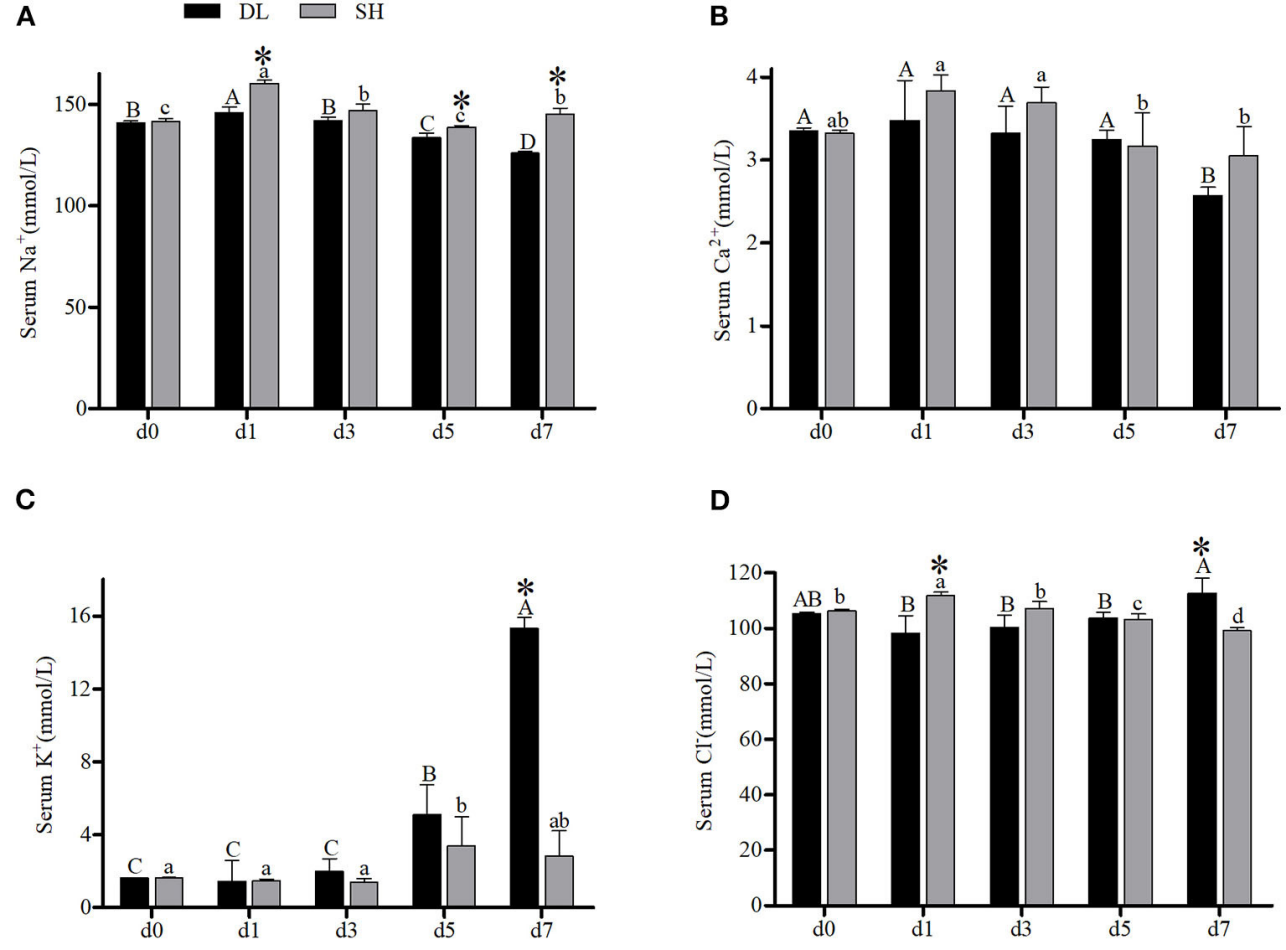
Serum ion concentrations (*n* = 9) of DL (the alkali form introduced from Lake Dali Nor) and SH (the freshwater form introduced from Songhua River) fish forms acclimated to AW (alkaline water) for 7 days. Different uppercase letters indicate significant differences within DL, and different lowercase letters indicate significant differences within SH. The asterisk (*) denotes a significant difference in serum ionic levels found between DL and SH exposed to bicarbonate AW.

The effects of the bicarbonate AW affect serum-ion levels of the DL and SH significantly as shown in Figure 1. When compared with d0, Amur ide that exposed to the bicarbonate AW affected their serum-ion levels. For the DL form, when exposed to bicarbonate AW showed that serum Na^+^ increased significantly at d1, then decreased significantly thereafter at d3, d5, and reached the lowest level at d7 (Figure 1A). Whereas for the SH form Amur, serum Na^+^ also increased significantly at d1 compared d0 (control group), then the serum Na^+^ decreased remarkably at d3 and reached the lowest level at d5 and then increased at d7. Overall, the effect of bicarbonate stress was remarkably affected by serum Na^+^ and resulted in serum Na^+^ fluctuations in both forms of Amur ide at d1, d5, and d7 (Figure 1A). A similar fluctuation trend of serum Ca^2+^ was found in both forms of Amur ide with progressively declining to the lowest levels at d7 under bicarbonate stress. Yet no significant difference in serum Ca^2+^ was detected between the fish forms (Figure 1B).

In contrast, an increasing trend of serum K^+^ was noticed in both forms of Amur ide (Figure 1C). In DL, the serum K^+^ was noticed to increase at d5 and reached the highest level at d7. A similar trend was observed in SH with their serum K^+^ increased significantly to the highest level at d5, but decreased at d7 (Figure 1C). A significantly different level of serum K^+^ between DL and SH was observed at d7. Interestingly, serum Cl^−^ showed a completely different pattern between DL and SH in response to bicarbonate stress (Figure 1D). For DL, serum Cl^−^ remained insignificantly different from d1, d3, and d5 compared to d0, but increased at d7 (Figure 1D). Differently in SH, higher serum Cl^−^ was noticed at d1 and then decreased progressively at d3, d5 and reached the lowest level at d7. A significant difference in serum Cl^−^ between DL and SH was recorded at d1 and d7 (Figure 1D).

### Gill NKA Activity

**Figure 2:**
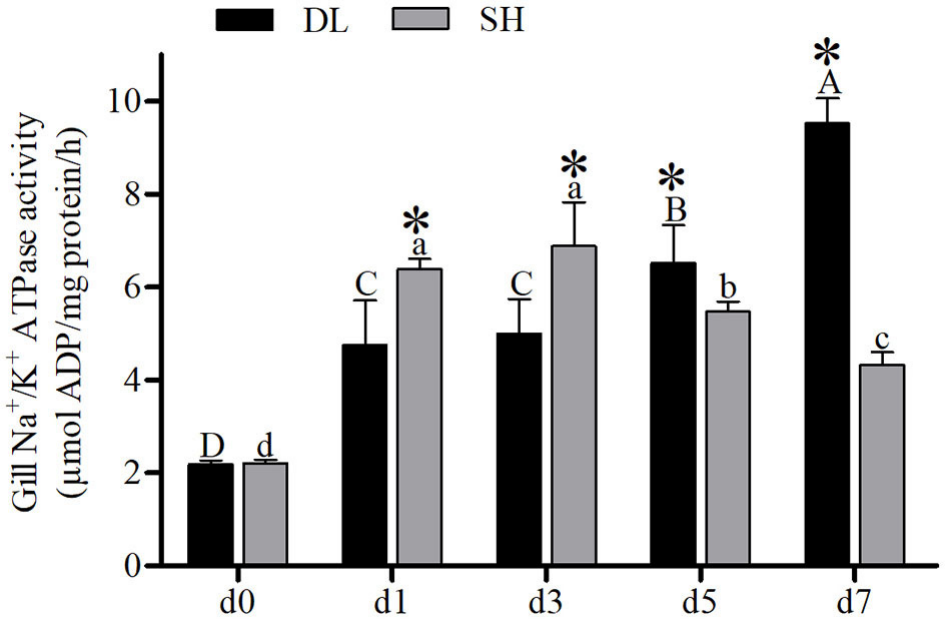
Gill NKA activity (*n* = 9) of DL (the alkali form introduced from Lake Dali Nor) and SH (the freshwater form introduced from Songhua River) fish forms acclimated to AW (alkaline water) for 7 days. Different uppercase letters indicate significant differences within DL, and different lowercase letters indicate significant differences within SH. The asterisk (*) denotes a significant difference found in NKA activity between DL and SH exposed to bicarbonate AW.

The enzyme activities of NKA for DL and SH showed a completely different pattern after being exposed to bicarbonate AW. At d0, the NKA activity level in both DL and SH exhibited a similar level (Figure 2). With prolonged bicarbonate exposure, the gills' NKA activity was found to increase at d5 and reached the highest level at d7 in DL. Whereas, the gills' NKA activity in the SH was expressed in a variable pattern. The SH gills' NKA activity decreased at d5 and continued to decrease to the lowest level at d7. In comparison between DL and SH, the gills' NKA activity levels were found significant at the different time-court intervals with higher gills' NKA activity found in SH compared to DL at the early stage of exposure to bicarbonate AW at d1 and d3. While higher gills' NKA activity was noticed at d5 and d7 in DL compared to SH at later-stage exposure (Figure 2).

### NKA mRNA Levels

**Figure 3:**
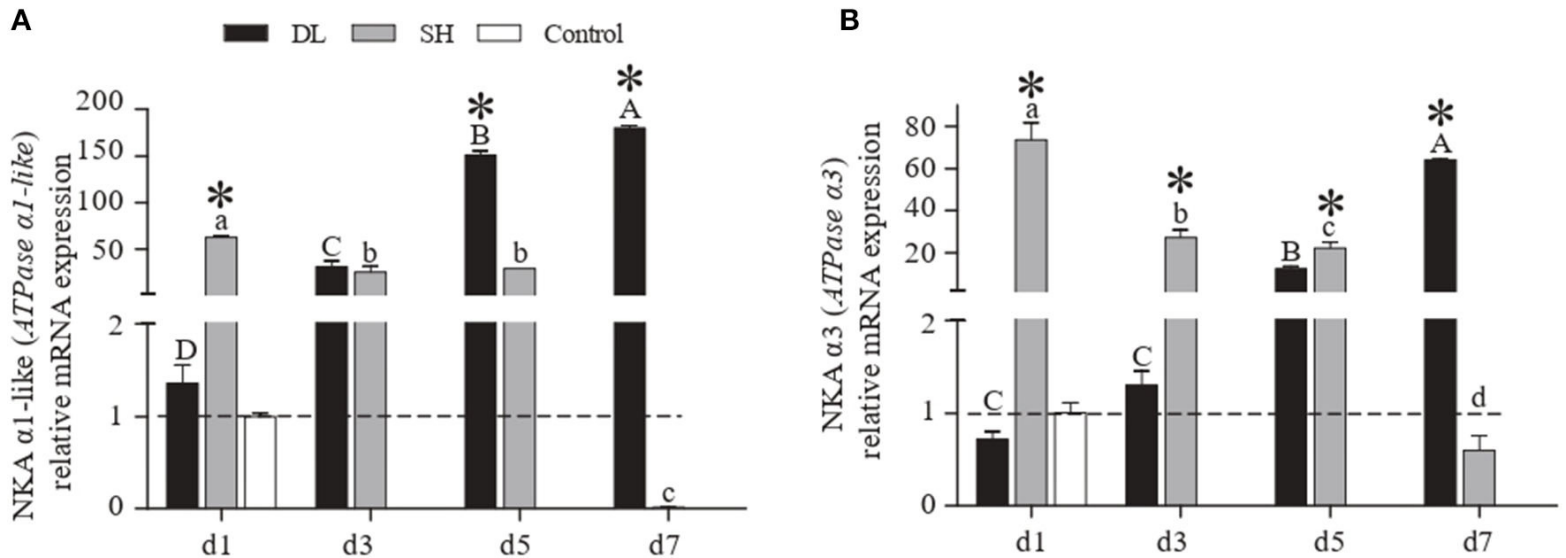
Gill NKA α1-like (*ATPase* α*1-like*) and NKAα3 (*ATPase* α*3*) mRNA levels (*n* = 3) for DL (the alkali form introduced from Lake Dali Nor) and SH (the freshwater form introduced from Songhua River) fish forms. Different uppercase letters indicate significant differences within DL, and different lowercase letters indicate significant differences within SH. The asterisk (*) denotes a significant difference found in the NKA α1-like or NKA α3 mRNA levels between DL and SH exposed to bicarbonate AW.

The patterns of mRNA NKA α1-like (*ATPase* α*1-like*) and NKA α3 (*ATPase* α*3*) expression in gill were similar to the NKA activity recorded in DL and SH (Figures 3A,B). Specifically, as the duration of bicarbonate AW exposure increased, both the α1-like and α3 mRNA levels increased rapidly and reached to highest levels at d7 for DL, which were 133 and 89 times higher than the levels at d1. In contrast to DL, both the α1-like and α3 mRNA levels in the SH form increased significantly at d1, then decreased to the lowest levels at d7 with was 6,285 and 123 times lower than d1, respectively. The DL can initiate and maintain higher expression levels of the NKA α-subunit mRNA at both d5 and d7 when compared to SH.

### NHE mRNA Levels

**Figure 4:**
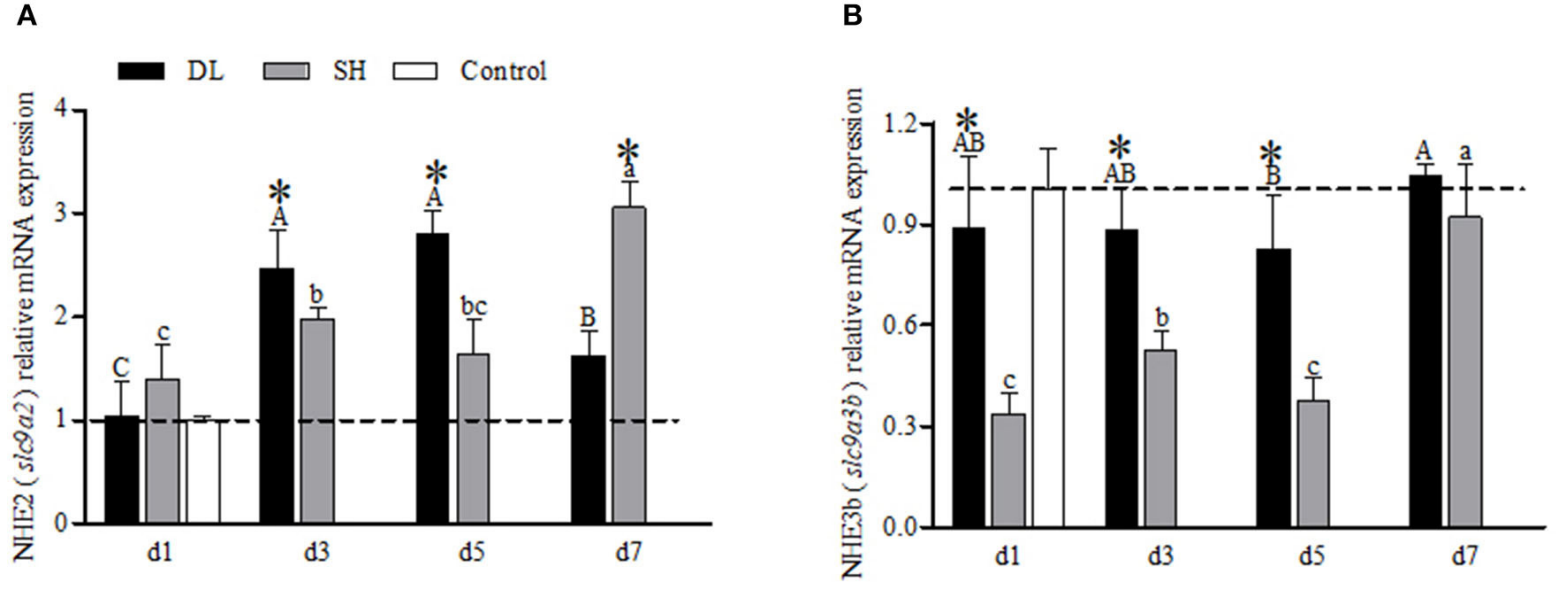
Gill NHE2 (*slc9a2*) and NHE3b (*slc9a3b*) mRNA levels (*n* = 3) for DL (the alkali form introduced from Lake Dali Nor) and SH (the freshwater form introduced from Songhua River) fish. Different uppercase letters indicate significant differences within DL, and different lowercase letters indicate significant differences within SH. The asterisk (*) denotes a significant difference found in the NHE2 or NHE3b mRNA levels between DL and SH exposed to bicarbonate AW.

The transcriptional response of NHE2 (*slc9a2*) in the gills expressed differently between the forms when exposed to bicarbonate AW (Figure 4A). In DL, initially, the NHE2 mRNA expression increased significantly to reach the highest level at d5 and decreased at d7 (Figure 4A). Whereas in SH, the NHE2 mRNA levels were found to increase significantly at d3 and d7 (Figure 4A). Except at d1, the NHE2 expression levels were significantly different at d3, d5, and d7 between DL and SH. Nevertheless, the NHE3b (*slc9a3b*) mRNA levels for DL were found relatively stable, only d5 was found lower compared to d7. For SH, the NHE3b mRNA expression level was found significantly higher at d7. In comparison between DL and SH, the NHE3b mRNA expression levels were found significantly different at d1, d3, and d5, respectively (Figure 4B).

### SLC4 mRNA Levels

**Figure 5:**
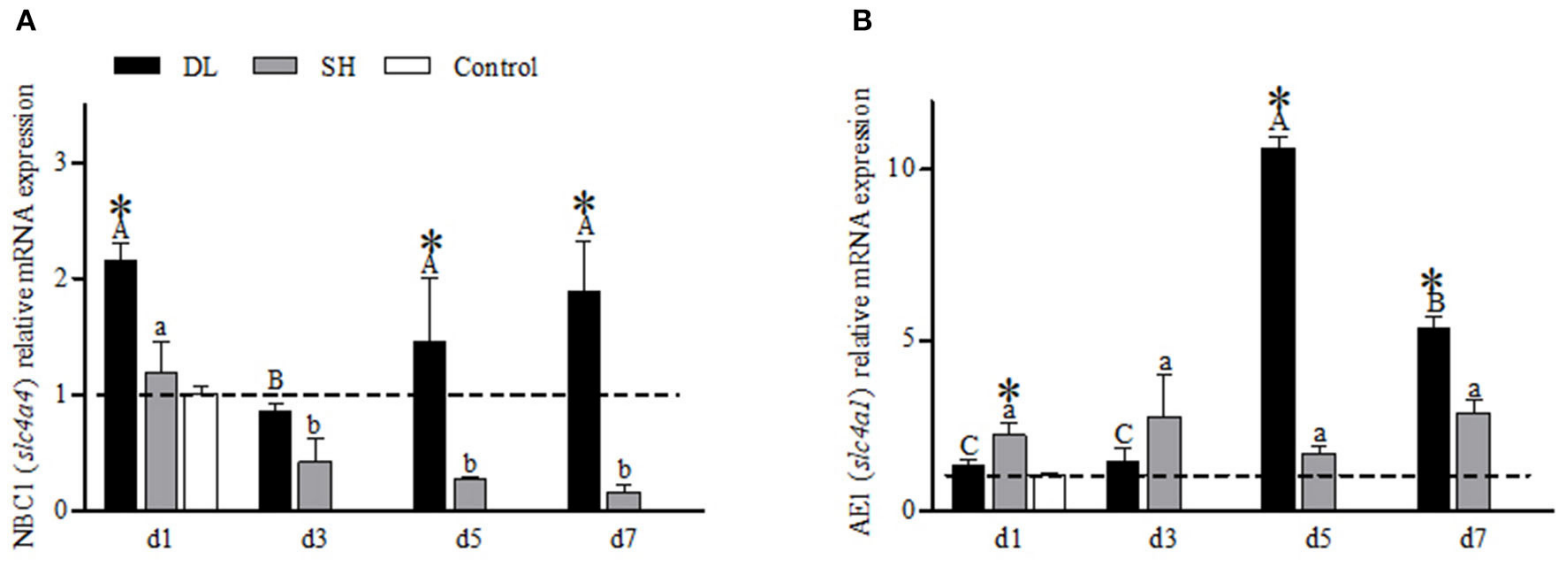
Gill NBC1 (*slc4a4*) and AE1 (*slc4a1*) mRNA levels (*n* = 3) for DL (the alkali form introduced from Lake Dali Nor) and SH (the freshwater form introduced from Songhua River) fish forms. Different uppercase letters indicate significant differences within DL, and different lowercase letters indicate significant differences within SH. The asterisk (*) denotes a significant difference found in the NBC1 or AE1 mRNA levels between DL and SH exposed to bicarbonate AW.

NBC1 (*slc4a4*) mRNA showed a differential expression pattern in DL and SH (Figure 5A). For DL, the NBC1 (*slc4a4*) mRNA levels were significantly expressed at d1, d5, and d7, while d3 was recorded at the lowest level, relative to the control fish. Meanwhile, for SH, a declining NBC1 mRNA expression level was found when exposed to bicarbonate AW (Figure 5A). The AE1 mRNA expression levels differed significantly between DL and SH on all days sampled, except d3 (Figure 5B). The highest expression of AE1 mRNA was noticed at d5 and decreased at d7 but remained higher compared to d1 and d3 were observed in DL. In contrast, the expression AE1 mRNA levels in SH remained relatively stable.

### SLC12 mRNA Levels

**Figure 6:**
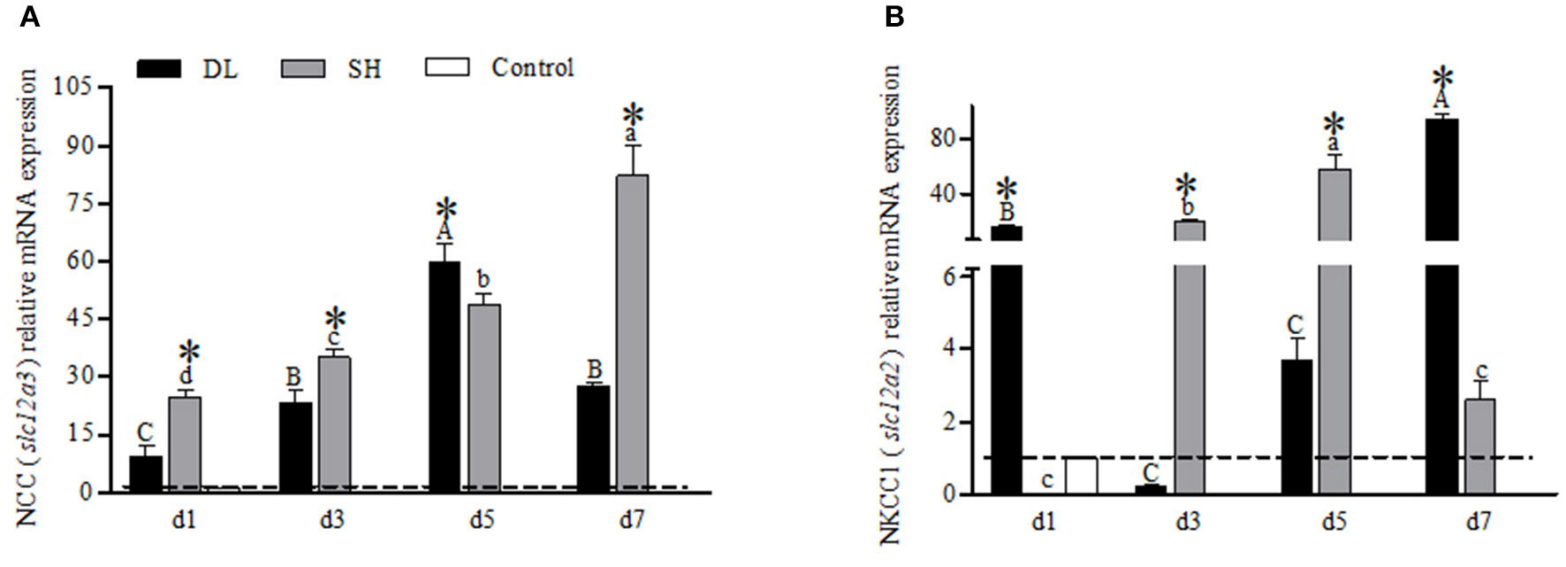
Gill NCC (*slc12a3*) and NKCC1 (*slc12a2*) mRNA levels (*n* = 3) for DL (the alkali form introduced from Lake Dali Nor) and SH (the freshwater form introduced from Songhua River) fish forms. Different uppercase letters indicate significant differences within DL, and different lowercase letters indicate significant differences within SH. The asterisk (*) denotes a significant difference found in the NCC or NKCC1 mRNA levels between DL and SH exposed to bicarbonate AW.

The transcriptional responses of NCC (*slc12a3*) and NKCC1 (*slc12a2*) in the gills of DL and SH were expressed differently when exposed to bicarbonate AW (Figure 6). For DL, the NCC mRNA expression levels were found significantly increased to the highest level at d5, and at d7, the level was returned to a level similar to d3 (Figure 6A). Whereas for SH, the NCC mRNA expression pattern was found significantly increased over the time course intervals. Accordingly, the expression response of NCC mRNA was found significantly different between DL and SH at all time-course intervals (Figure 6A). Contradictory, the NKCC1 mRNA expression in DL was noticed significantly higher at d1 and dropped to a level lower than the control group at d3, but thereafter the NKCC1 mRNA levels were found increased and reached the highest level at d7. Unlike DL, the NKCC1 mRNA expression levels were found significantly higher at d3 and d5, followed by a significant decline at d7. The expression levels of NKCC1 mRNA between DL and SH were significantly different at all times point (Figure 6B).

### SLC26 mRNA Levels

**Figure 7:**
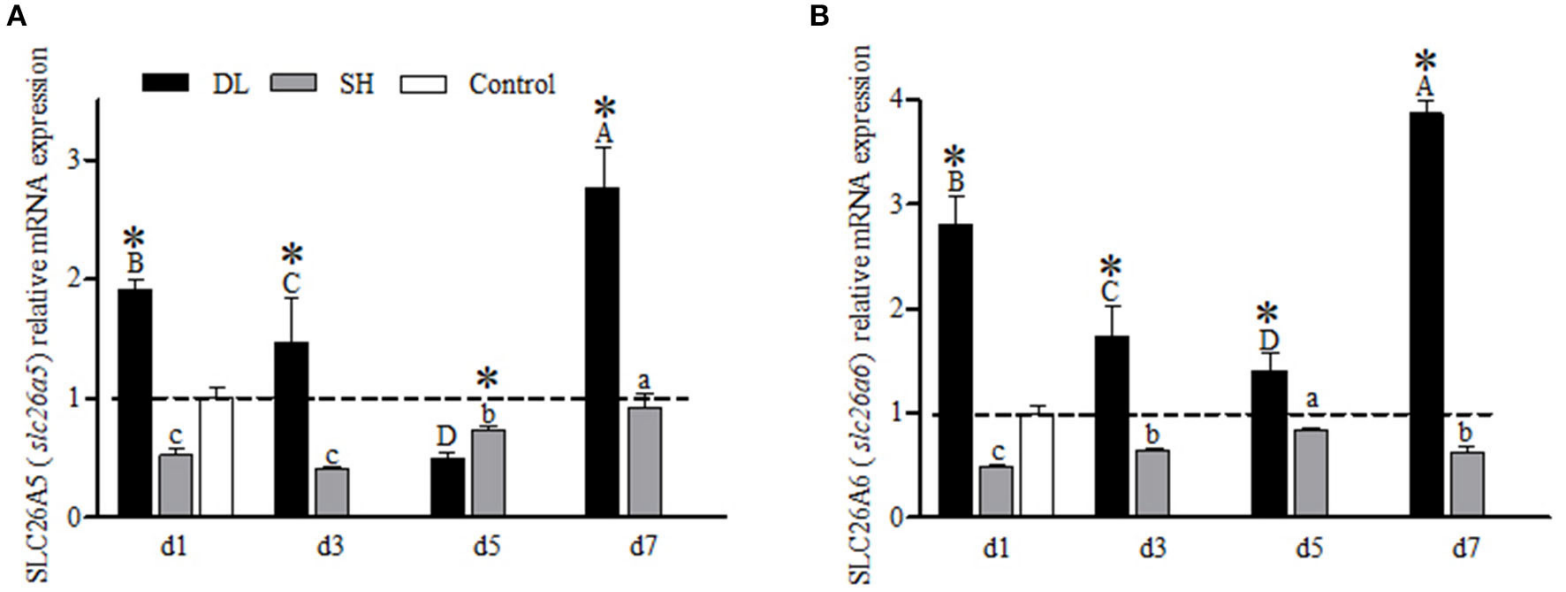
Gill SLC26A5 (*slc26a5*) and SLC26A6 (*slc26a6*) mRNA levels (*n* = 3) for DL (the alkali form introduced from Lake Dali Nor) and SH (the freshwater form introduced from Songhua River) fish forms. Different uppercase letters indicate significant differences within DL, and different lowercase letters indicate significant differences within SH. The asterisk (*) denotes a significant difference found in the SLC26A5 or SLC26A6 mRNA levels between DL and SH exposed to bicarbonate AW.

For the gill SLC26A5 (*slc26a5*) and SLC26A6 (*slc26a6*) mRNA expression levels showed a similar trend in DL with decreasing trend at d3 and d5 but increased to a higher level at d7 as compared to d1 (Figures 7A,B). For SH, both SLC26A5 and SLC26A6 expression levels were lower than the control at all exposure time points. However, when comparing DL and SH, the SLC26A5 and SLC26A6 expression levels were found significantly different at all time-course intervals (Figures 7A,B).

## Discussion

The Amur ide living in an alkaline environment is a well-documented phenomenon, with much research carried out to understand how this fish has been able to maintain its fitness in an extremely AW habitat *via* phenotypic adaptation or genetic evolution (Geng and Zhang, 1988; Chi, 2010; Chang et al., 2013b, 2014; Xu et al., 2017; Wang et al., 2021; Zhao et al., 2021). Research investigating how Amur ide performs physiologically adaptability to extreme alkaline condition remained to be investigated. Therefore, this experiment was conducted to identify the physiological and molecular differences to address how the alkali form DL can adapt to AW rapidly, while in contrast to the freshwater-form SH when exposed to bicarbonate stress. Our results showed that alkali-form DL transferred from FW to AW initiated key ion-regulation responses to maintain Na^+^ and acid-base balance. These responses were physiologically relevant at the early-exposure stage d3 (72 h), later followed by related genes upregulation triggering molecular response mechanisms at d5 (120 h), whereas the freshwater-form SH responded at a later stage showing a long-term adaptation strategy.

### Ionic Regulation in the Alkali form of Amur Ide Living Under Alkaline Condition

The migration of euryhaline teleost fishes from FW into SW requires gill plasticity to change from an ion-absorbing tissue to an ion-secreting tissue known as functional ionocytes. In most of the euryhaline fishes, this ionocyte activation is associated with upregulation of gills' NKA activity (Bystriansky et al., 2007b). However, our result showed that when DL was exposed to AW, their gill transporter activities and mRNA were expressed differently. Similar to most of the other euryhaline teleosts, the alkali-form DL can upregulate the gills' NKA mRNA levels and activity when exposed to AW (Figures 2, 3). These increases in NKA mRNA levels were seen on day 3 and NKA activity on day 5 exposure to AW in the alkali form DL (Figures 2, 3). This response is similar to other euryhaline teleosts when they migrate from freshwater to saline environments. For example, when salmonids acclimated to SW for 11 days was reported with an increase of NKA α-mRNA followed by greater NKA activity (D'Cotta et al., 2000; Bystriansky et al., 2006). This may be due to the time required for *de novo* synthesis of the NKA protein that results in a time lag between the increase of mRNA expression and actual functional activity.

In contrast to the alkali-form DL, low NKA activity was noticed with NKA mRNA levels downregulation following the exposure of freshwater-form SH exposed to AW (Figures 2, 3). Previous studies have shown that an ecological transition to a completely new environment results in an evolutionary trade-off in osmoregulatory functionality (Velotta et al., 2014, 2016). This is paralleled with the reduction of NKA mRNA expression levels consistently found for landlocked freshwater fish compared to its ancestral seawater or anadromous forms at all salinities (Bystriansky et al., 2007a; Nilsen et al., 2007; Lee et al., 2011a; Velotta et al., 2015). This is in agreement with our finding showing that an evolved stronger role of NKA ionic regulation in the alkali form of Amur ide than its ancestral freshwater form to cope with the alkalinity challenge.

**Figure 8:**
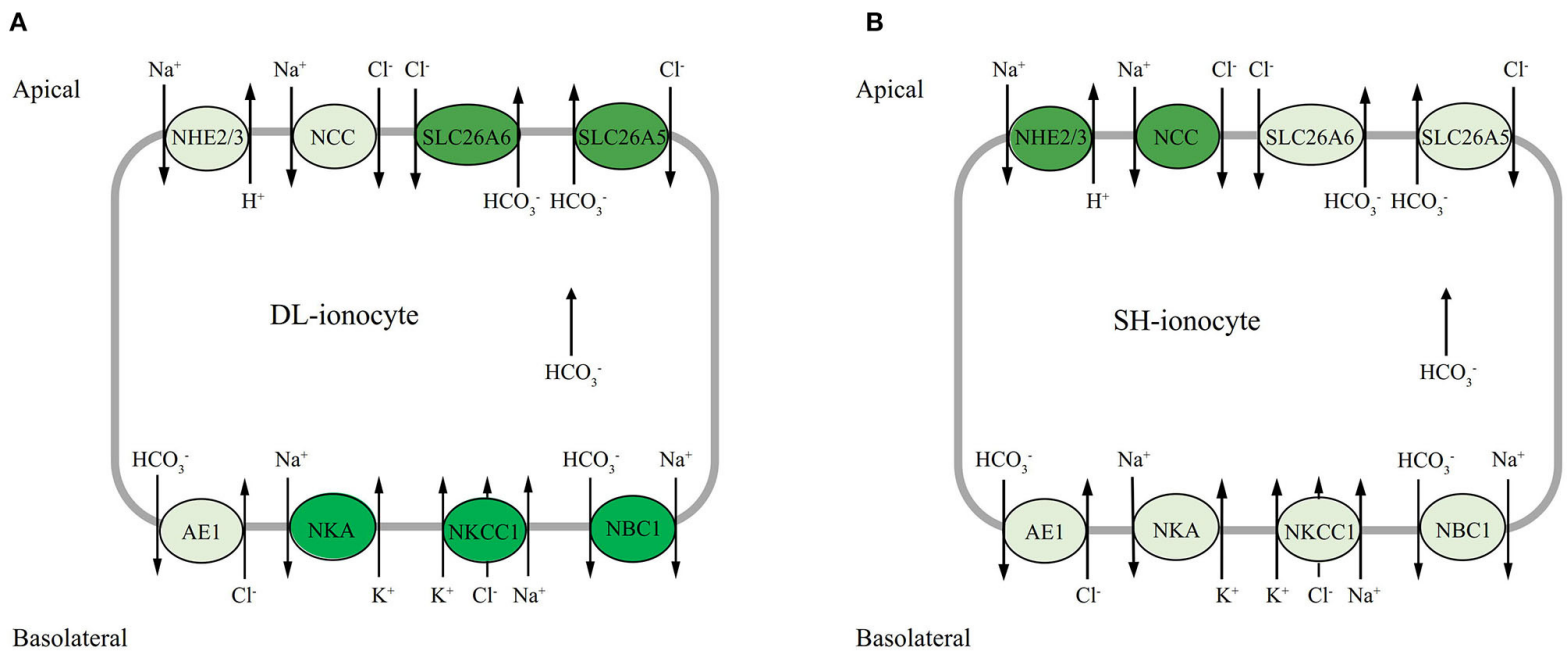
Hypothetical model of mRNA expression of each gene in the gills of DL (the alkali form introduced from Lake Dali Nor) and SH (the freshwater form introduced from Songhua River) fish forms in 50 mM alkaline water. The dark background denotes upregulated expression, whereas the light background denotes downregulated expression.

Na^+^/K^+^ ATPase may participate in both ion uptake and salt excretion in the teleost gill depend on location, surrounding condition, and specific species. The early investigation noted that NKA immunoreactivity is present basolaterally in ionocytes of both freshwater- and seawater-acclimated fish, making it difficult to ascertain the actual functioning of ion absorption in FW and salt secretion in SW (Hiroi and Mccormick, 2012). Nevertheless, most of the evidence to date suggests that NKA may work with apical transporters such as NHE2 or/and NHE3 and NCC, and also basolateral transporters like NKCC1 and NBC1, to accomplish transepithelial ion transport functions (Hiroi and Mccormick, 2012; Michael et al., 2016; Lewis and Kwong, 2018). According to our results, the expression profiles of both apical Na^+^ transporters NHE2 and NCC were highly correlated, where both transporters increased to the highest levels at d5 and decreased at d7 in the alkali-form DL. This suggests that when DL was exposed to high bicarbonate AW (NaHCO_3_), the activities of these two transporters were affected by the alkaline condition (Figure 8). Interestingly, in the alkali-form DL, we found that the expression of basolateral Na^+^ transporters NBC1 was also tightly correlated. Evidently decreased at d3 and increased at d5 and d7 which was believed to facilitate Na^+^ absorption in the alkali-form DL (Figure 8). This scenario is consistent with the trend of a significant decrease in the overall Na^+^ concentration in serum of the alkali-form DL (Figure 1). Previous studies on tilapia have shown that the “freshwater-type” cotransporter NCC is involved in ion absorption, whereas the “seawater-type” cotransporter NKCC1 is involved in salt secretion (Hiroi et al., 2008; Hiroi and Mccormick, 2012). Using the specific NBC1 antibody of Atlantic cod (*Gadus morhua*), NBC1 and NKA were found colocalized on the basolateral membrane of branchial ionocytes (Michael et al., 2016). This view is consistent with our results shown that the mRNA expression of NCC increased at all time-course points in the freshwater-form SH exposed to bicarbonate stress. Thus, together with the mRNA expression patterns of gill transporters and serum ionic concentrations allow us to note that Na^+^ may enter the branchial cells *via* the NCC, NKCC1, and NHE2 transporters in the alkali form of DL upon bicarbonate exposure. Then the Na^+^ would be transported out of *via* NBC1 activity driven by the Na^+^ gradient and NKA activity (Figure 8). Further studies are nonetheless needed to clearly define the cellular colocalization of these vital transporters in the alkali form of DL.

### Acid-Base Regulation in the Alkali Form of Amur ide Exposed to Alkalinity Challenge

The compensatory effect of gill cells in teleost does not only involve net fluxes acid or base secretions but also is accompanied by an exchange of equivalent ions, such as Na^+^, Cl^−^, and ${\text{NH}}_{4}^{+}$, directly or indirectly to achieve body-fluid ionic and acid-base homeostasis (Hwang and Perry, 2010; Hwang and Chou, 2013). Table 2 shows that under alkaline conditions the blood pH in the alkali form of Amur ide fish was relatively stable when exposed to bicarbonate AW. This suggests the alkali form of Amur ide has evolved a strong ability to secrete ${\text{HCO}}_{3}^{-}$ to avoid incurring alkalosis while still effectively maintaining its acid-base balance to live under extreme AW.

The two bicarbonate transporter families—SLC26 (SLC26a5 and SLC26a6) and SLC4 (AE1 and NBC1) are involved in transport ${\text{HCO}}_{3}^{-}$ across the gill epithelium, either on the apical side in exchange for Cl^−^ (transporter family SLC26) (Perry et al., 2009; Boyle et al., 2015) or basolaterally into the plasma (transporter family SLC4) (Tang and Lee, 2007; Wang et al., 2009; Lee et al., 2011b; Esbaugh et al., 2012). It is interesting to highlight the bifunctional role of ${\text{HCO}}_{3}^{-}$ secretion in the two contrasting forms of Amur ide. In the alkali form (Roessig et al.), SLC26A5 and SLC26A6 have similar mRNA expression patterns and were sensitive to alkalinity stress with transcriptional response was observed within 24 h exposure to AW. In addition, SLC26A5 and SLC26A6 mRNA were found upregulated at d7 under AW exposure with SLC26A6 outperformed than SLC26A5. In contrast, SLC26A5 and SLC26A6 appeared to be nonsensitive to alkalinity stress in the freshwater-form SH. This was proven with both SLC26A5 and SLC26A6 that mRNA levels remained lower than those of the control under AW exposure. The expression of NBC1 mRNA was found upregulated to day 3 in the alkali-form DL. In comparison with NBC1, AE1 mRNA had a different expression pattern, one that seemed inconsistent during the bicarbonate AW acclimation, but still maintains a higher expression level. On the whole, it would appear that SLC26A5, SLC26A6, and NBC1 are correlated in the present study; however, these bicarbonate transporters may be located in different ionocytes. This is in line with another member of the SLC26 family, known as pendrin in that the SLC26A4 found in Atlantic stingray (*Dasyatis sabina*) was clearly located in the H^+^ ATPase-rich cells and not in the NKA-rich cells (Piermarini et al., 2002). Other studies have confirmed that NBC1 is colocalized with NKA in salmonids and demonstrated that SLC26 and NBC1 were located in different ionocytes (Hiroi and Mccormick, 2012). Moreover, some studies indicated that NBC1 coupled with NCC in the same ionocytes involves Cl^−^ and/or Na^+^ uptake functions in zebrafish (*Danio rerio*) (Wang et al., 2009) and Mozambique tilapia (*Oreochromis mossambicus*) (Inokuchi et al., 2008). Considering mRNA expression profiles found in the study, as well with increased serum Cl^−^ and decreased in Na^+^ concentrations, we suggest that it is reasonable that NCC is located at the apical membranes, while NBC1, NKCC1, and NKA are presented at basolateral membranes coupled with other transporters (SLC26) and enzymes to achieve the transepithelial Cl^−^, Na^+^ uptake/base secretion function in the alkali-form DL exposed to bicarbonate AW (Figure 8). Nevertheless, this proposed model awaits further investigation to confirm the localization of these ionocytes using a rigorous immunohistochemistry approach.

## Conclusions

Amur ide is an excellent model to understand the physiological and genetic basis of alkaline adaptation through an evolutionary lens. Here, we investigated differences in blood pH and serum key ions, gills' NKA activity, and gill transporters (NHE2/3, NBC1, AE1, NCC, NKCC1, SLC26A5, and SLC26A6) mRNA expression to alkalinity adaptation between two forms of Amur ide fish differing their alkalinity tolerance. Based on our results, we propose the alkali form of Amur ide may have evolved a strong ability to maintain ionic and acid-base balancing *via* basolateral NKA with NBC1, and apical ionic transporters, especially NCC to regulate Cl^−^ and Na^+^ uptake/base secretion functions for ion homeostasis. In addition, the bicarbonate transporter SLC26 may play a pivotal role in modulating the extrusion of bicarbonate under alkaline conditions. Therefore, we conclude that the alkali form of Amur ide acclimates more quickly and effectively to bicarbonate AW than freshwater form.

## Data Availability Statement

The original contributions presented in the study are included in the article/Supplementary Material; further inquiries can be directed to the corresponding author(s).

## Ethics Statement

The animal study was reviewed and approved by The Laboratory Animal Management Committee Heilongjiang River Fisheries Research Institute, Chinese Academy of Fishery Sciences.

## Author Contributions

YC and LQL conceived the study. XZ, BS, LL, and LZ performed the research. XZ and SW analyzed the data. YC wrote the manuscript. HL revised the manuscript. All authors reviewed and approved the submitted version.

## Funding

This study was supported by the National Key R & D Program of China (Grant No. 2019YFD0900405); the National Natural Science Foundation of China (Grant No. 31602136); the Natural Science Foundation of Heilongjiang Province of China (Grant No. C2016070); and the Central Public-interest Scientific Institution Basal Research Fund, CAFS (Grant Nos. 2017 HY-ZD0404; 2019ZD0601, 2020TD22).

## Conflict of Interest

The authors declare that the research was conducted in the absence of any commercial or financial relationships that could be construed as a potential conflict of interest.

## Publisher's Note

All claims expressed in this article are solely those of the authors and do not necessarily represent those of their affiliated organizations, or those of the publisher, the editors and the reviewers. Any product that may be evaluated in this article, or claim that may be made by its manufacturer, is not guaranteed or endorsed by the publisher.
